# Supplementary material for: Expression profiles of TRPV1, TRPV4, TLR4 and ERK1/2 in the dorsal root ganglionic neurons of a cancer-induced neuropathy rat model
Source: PeerJ. 2018 Apr 4;6:e4622. doi: 10.7717/peerj.4622 (PMC5889703; doi:10.7717/peerj.4622)

Expression Profiles of TRPV1, TRPV4, TLR4 and ERK1/2 in the Dorsal Root Ganglionic Neurons of a Cancer-Induced Neuropathy Rat Model

Figure 4:

- Anti-TRPV1 antibody

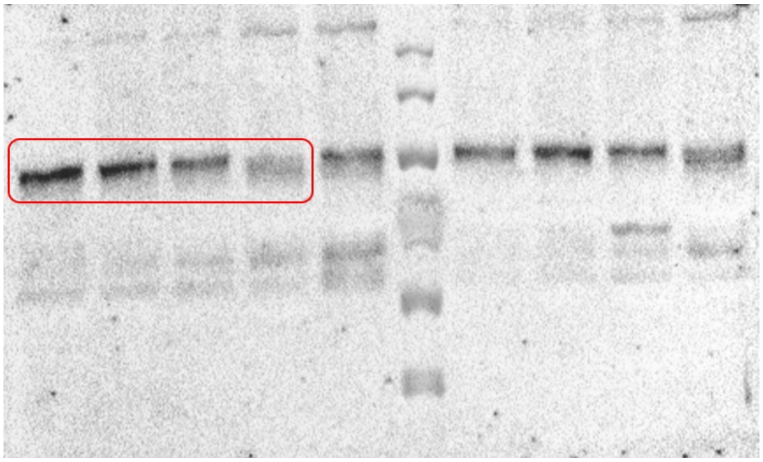

- Anti-β-actin antibody

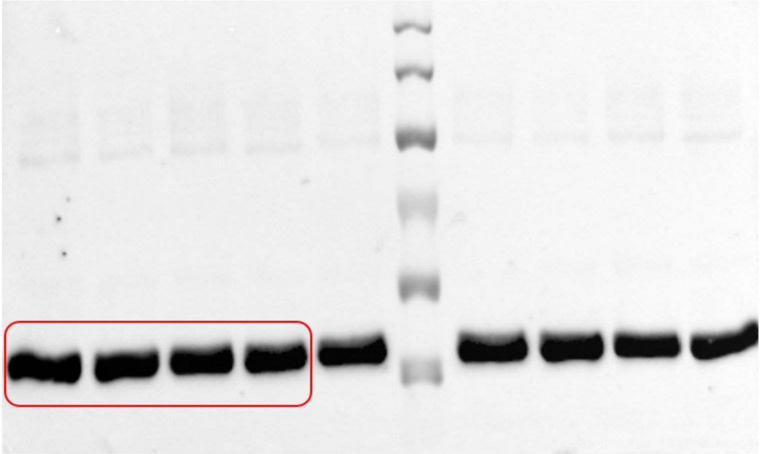

Figure 7:

- Anti-TRPV4 antibody

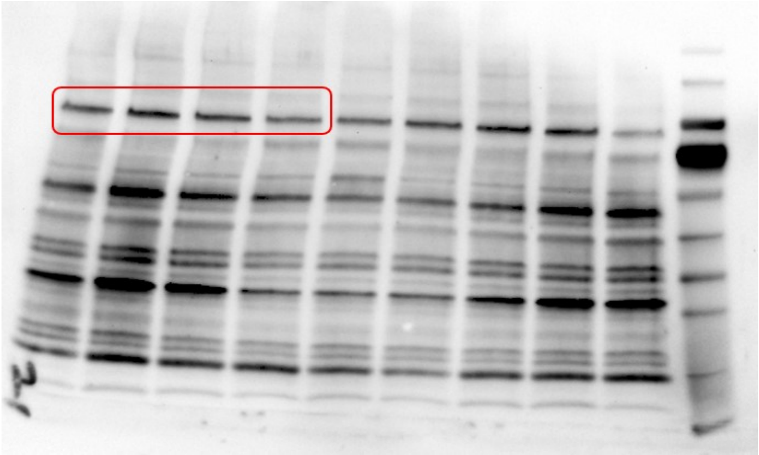

- Anti-β-actin antibody

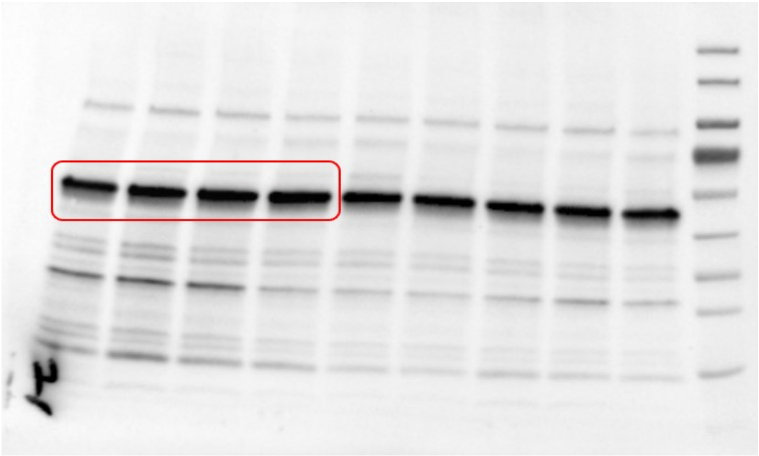

Supplement: Supplemental Information 1 — Original Western blot images for TRPV1 and TRPV4 and beta-actin. [file peerj-06-4622-s001.pdf]
